# Extra Supplement.—The Nursing Mirror

**Published:** 1888-11-03

**Authors:** 


### The Nursing Mirror.

All Contributions for this Supplement should be addressed "The Nursing Mirror," care of The Editor, 38, Old Jewry, E.C.

### En Passant.

BIRMINGHAM INFIRMARY.—The following is from a local account of this infirmary: "Great inconvenience having been experienced by nurses when off duty being distributed in various parts of the institution, it was decided to erect a nurses' home a short distance away from the main building. This stands at the rear, and is more highly finished than the other part of the work. Here the matron and assistant-matron have their rooms, and there are seventy-two bed-rooms for the nurses. These latter are all of a uniform size and are similarly fitted up. There is a large room in which the nurses can associate together, and in which the new matron (Miss Gibson), who is, perhaps, the most accomplished nurse in England, will give her lectures. Miss Gibson hails from the Liverpool Infirmary, and has been trained under the Nightingale system. At the back of the Home the formation of a tennis lawn and grounds for recreation is in contemplation."

REGISTRATION OF MIDWIVES. — The meeting held at Oxford on October 24th, to discuss the registration of midwives, was most interesting and successful. A most distinguished company assembled, including three members of the Midwives Institute; Miss Davis, of the Maternity Hospital, Manchester; Miss Deniston, superintendent of the Acland Home; Mrs. Burdon Sanderson; Mrs. A. Vernon Harcourt; Mrs. R. Poole; Mrs. Merry, of Lincoln College; and many others. Two papers were read, and were followed by quite a lively discussion, and the proceedings terminated by many of those present signing the petition in favour of midwives' registration. The "poor neighbour" difficulty came to the fore as usual, many ladies insisting on believing that the "poor neighbour" is quite capable to undertaking duties which should always be left to a trained nurse or a doctor, and regretting the loss of employment to these same poor neighbours, who, in the event of registration, would probably secure other employment more suited to their capabilities. It was stated by a midwife that she earned £200 a-year, though her fee was only ten shillings.

NURSES AND PRESENTS.—We have had a letter purporting to be from a lady who is hurt, because the rules of the Rivers Street Institute, Bath, do not allow presents to be accepted by nurses without special permission from the superintendent. The writer says the nurses are paid £20 a-year, and earn two guineas a-week, which is, unluckily, a fact applicable to most of these institutions. Then she goes on to state that often a nurse ruins her health in attending on some patient who can make no return for benefits received; and as for the Institute, it only cares for a sick-nurse for a month or six weeks; and then if she does not get better, sends her to a hospital, or discharges her. Very properly the writer does not approve of these proceedings, and asks that Rule 12 of the Institute may be repealed; that grateful patients may be allowed to subscribe to the Pension Fund for Nurses, and to give small gifts, not in money, to any nurse who has cared for them. The following is a rule of the Ipswich Nurses' Home: "A nurse may not receive any gratuity, other than a book, or other article of small value, which may be given without infringement of this rule. Any further offering, in gratitude for her services, is to be given to the institution," and is one common to the better class of institutions. We should be glad to have the opinions of nurses on this vexed question; also of those who have been patients. The feelings of both should be consulted in the framing of rules with regard to presents.

BRIGHTENING HOSPITAL LIFE.—A concert was given to the nurses and patients of the Royal Free Hospital, Gray's Inn Road, last week, by Mr. F. W. Speaight, Chairman of the Saturday Evening Free Entertainment, St. Alban's Hall, Holborn. Signor Tartaglioni and several of his pupils were amongst those who kindly assisted, and whose efforts were warmly appreciated by an attentive audience.

PROPER ROOMS FOR NURSES.—The committee of the Royal Southern Hospital have found it necessary to increase the accommodation at this institution, and are taking steps to provide additional premises, which are much needed. The mortuary and post-mortem rooms are within the hospital buildings, and this is entirely condemned by all authorities on such subjects. For some time the accommodation for the nursing staff under the system of nursing now considered essential has proved insufficient. As a temporary measure one of the unused wards was fitted up as a dormitory, but this could only be considered a makeshift, and it has, besides, been found in that in large hospitals it much conduces to the health and consequent efficiency of the nurses that their dormitories should be in quarters separate from the building containing the hospital wards. Improved accommodation is much needed for the nursing institution attached to the hospital, which has proved of great value to those requiring trained private nurses in their homes. The nurses of this institution when off duty are now located in a house in Grafton Street; but this arrangement has many disadvantages, and it would be well to have the whole nursing establishment under one roof. To remedy these defects the committee propose to erect outside the present building, and on land adjoining the hospital, a mortuary and post-mortem room, and also a nurses' house, in which will be provided dormitories for the nursing staff, apart from the wards, and a home when off duty for the private nurses of the institution affiliated to the hospital. It is estimated that the cost of the land and buildings will be about £7,000.

BORN NURSES.—A. E. S. writes to us under this heading as follows: "It is refreshing to find people are beginning to look on 'born nurses' as much of a myth as 'born cooks or born dress-makers.' We sincerely trust Miss Morton's book will fall into the hands of the numberless romantic girls who are anxious to go in for nursing, or who are such a trial to those who have the training of these so-called 'born nurses.' Training is real hard, dirty work, far rougher than the generality of servants would submit to in these days—to say nothing of the long hours. So many professionals on first entering a hospital appear to think all work menial that does not consist in personal attendance on a patient; they do not consider that sweeping, cleaning, doing up fires is bound to be done by someone. We ourselves would much prefer the cleaning of fire-sides or sinks to the putting to bed of the many drunken men and women who, sad to relate, fall to the lot of all nurses. What we want is thorough hard-working professionals (ladies or not), with no nonsense, no romantic ideas, who have no thought (during working hours) but their work, who take a pride in making and keeping everything, themselves included, spotlessly clean, who would be ashamed to let work remain undone because it was not exactly theirs. Surely it is high time that all the romantic stuff talked about hospital nursing should cease; that we should be fully recognised as women, determined to do our very best in whatever we may engage, be it nursing a 'bad case,' cleaning a sink, or taking care that all food is well cooked and made to go as far as possible, knowing we are all only stewards. We have each our own particular reason for the life we have chosen, but let us trust we have heard the last of a girl applying for the place of probationer because she, or her foolish friends, think her a 'born nurse.'"

### Letters from a Probationer.

No. V.—Operation Day.

Dear ——, Though I am rather tired to-night, I must scribble you a few lines, according to your request, though, had you known this was the day for operations, I am sure you would not have asked it. Of course there are often small operations going on in the hospital, but so far as possible all the principal ones are kept for Fridays, when two celebrated surgeons attend. It is the busiest day of the week, now I am moved to the surgical side, and also the most trying. To-day I spent nearly three hours in the operating theatre, and saw seven operations performed. Perhaps I ought not to say "saw," for in many of the cases I was too busy attending to my own duties to do more than just glance at the surgeon's work. But one operation I followed closely, for the surgeon gave a sort of little lecture on it first and drew a diagram on the black-board of what he meant to do. I watched him anxiously. It was a case of disease of the jawbone, and surgeons and students were all so intent on the operation that it was Sister who first touched the house-surgeon's arm and whispered to him that the blood was getting into the man's throat and choking him. I ought to explain to you that the operating-room consists of a semicircular arena, and three rows of seats stretching up and back, very like our lecture-hall at school, and about the same size. A table, of marvellous construction, occupies the middle of the arena, and on this the patient is placed. With a touch of the hand the surgeon can raise or lower any portion of this table in a moment. The seats are generally pretty full of students, who are quiet and attentive enough while an operation is going on, but up to all sorts of larks between whiles. The surgeons, the dresser to the case, and the nurse are the only people allowed in the arena. I suppose I must not describe to you any further details, for they sound so ghastly to outsiders; but you must understand the patient does not see them: he is put under an anæsthetic in a dingy little ante-room, and only wheeled into the great light theatre when he is unconscious. What are the mock tragedies you witness at the Lyceum or Haymarket compared with the real tragedies performed in the intense silence of our theatre? Compare the audience and the actors in each case, and pray that your acquaintance may be limited to the place of amusement. We nearly had a death under chloroform last week, but relays of students kept up artificial respiration for over three hours, and then the woman began to breathe naturally again. It is most extraordinary to think of, that that woman was practically dead for three hours, and is now alive. Recovery after such a long suspension of breathing is very uncommon, and we were all greatly excited about the case, and much relieved when the efforts of the students were at last successful. When we have an ovarian operation it is generally performed in a specially-prepared room, which has been thoroughly disinfected. The room is emptied and aired, and then door and window are closed, ventilators opened, a fire lit, and the carbolic spray set going. The table is prepared with a perfectly new mackintosh, and the bed is made up with new sheets, pillow-cases, &c., no effort or expense being spared to exclude the insidious germ. Only one or two advanced students are allowed to be present, and after the operation the spray is kept going for thirty hours. For my part, I hope I may never be "Nurse Operation;" her post is the most lonely, anxious, and wearisome one in the hospital, and yet many covet it. It is quite trying enough for me to have charge of a complicated fracture, diseased jaw-bone, or other minor case, and my heart generally goes double rate when I am marching behind the stretcher which bears one of my patients to the theatre. In time, though, I suppose I shall get used even to this. There! You won't ask me to write again on Friday, after this dismal epistle.

### Opening of a Nursing Institution at Taunton.

On October 25th the Hon. W. H. B. Portman opened the Victoria Jubilee Nursing Institute, which has been erected in connexion with the Taunton and Somerset Hospital at Taunton. The latter institution is a memorial of the Jubilee of the reign of George III., and it was a happy suggestion on the part of Dr. Edward Liddon, whose zeal for the welfare of the Hospital is so well known, to commemorate the Jubilee of Queen Victoria by extending the usefulness of the old institution. At a county meeting in 1887 the scheme was fully laid before the subscribers and general public, the cost of erecting and endowing the Institute being estimated at £10,000. This sum has actually been raised. The Institute, which is built principally of brick, and is of a classical style of architecture, adjoins the west wing of the Taunton and Somerset Hospital, and occupies the site of the former out-patients' department and a few cottages that had for a long time been in a dilapidated condition. On the key-stone over the doorway is carved the initials "J. B.," surrounded by a wreath of oak leaves, the initials being those of the munificent donor of £5,000 who has chosen that his name shall not be published. Over the key-stone is carved the date of the erection of the building, "1887." Flanking the entrance are six semi-circular headed plate-glass windows, three on each side, above each of which is a neat terra-cotta cornice. The key-stones of these windows are beautifully carved, and the first on each side of the door bear the initials of two other donors, Dr. Edward Liddon and Dr. William Marwood Kelly, encircled by wreaths of civic crowns of carved bay and olive leaves. Although the Institute has been built for the accommodation of young women desirous of becoming trained nurses, a portion of it is to be utilized for other important purposes, the whole of the floor on the street level being occupied by a suite of rooms devoted to out-patients, who have hitherto had to put up with a little inconvenience, owing to the want of a suitable place in the hospital to receive them. The ceiling of the out-patients' hall is panelled, and is of special design. The centre-piece is a magnificent piece of work, representing the Order of the Star of India, which encloses the monogram "V.R.I." Between the five points of the star (which are said to represent the number of decades of her Majesty's reign) the rose, the shamrock, and the thistle are to be introduced alternately. Encircling the Star of India is a larger star, with fifty points, referring to the years of her Majesty's reign at the time the Institute was built. The Nursing Institute proper occupies the two floors above the out-patients' department. These rooms will be connected with the hospital by a dull glass corridor on the basement at the back of the Nursing Institute. There will also be corridors at the level of the two upper storeys, so as to enable the nurses to pass with facility from the sick-wards of the old hospital to their own rooms, but the main staircase for general purposes will be one that opens from the glass corridor just alluded to. The contractor was Mr. William Templeman, of Eastreach, Taunton. The architect, from whose plans and under whose personal superintendence the whole of the work has been carried out, is Mr. Houghton Spencer, of Taunton.

### Presentation.

On Tuesday last, at the Middlesex Hospital, Dr. and Mrs. Bedford Fenwick presented Nurse Bayliss with a very handsome gold watch and chain, from the members of the British Nurses' Association. Nurse Bayliss has lately returned from Eastbourne.

### Everybody's Opinion.

[*Correspondence on all subjects is invited. No communications can be entertained if the name and address of the correspondent is not given, or unless one side of the paper only be written on.*]

THE BRITISH NURSES' ASSOCIATION.—I have read with great interest the opinions of Nurse and Matron in your last week's issue of THE HOSPITAL on the above Association. I am another, a Country Matron, who cannot at present see the benefit any trained nurse will derive in joining the British Nurses' Association. For what reason was it started? All women who are trained nurses will be compelled to be registered. Should a Charter ever be passed, Miss Wood kindly informed me "that country matrons and nurses would benefit by the sympathy of the Association." I consider a subscription of 10s. far too much money to become a member, unless one knows she will benefit by it, especially where one's salary will hardly allow one to save for the future. I, for one, intend saving all for the Pension Fund, and we, as a body, cannot be too thankful to those who first started it.—A COUNTRY MATRON.

ZENANA MISSIONS.—Miss Robinson kindly forwards us the following letter she has received:—" Mission School, Beyrout, Syria, Oct. 7th., Dear Miss Robinson, My medical work goes on increasing, and I only long for help to carry it out more fully. I must not omit to thank you for the very kind mention you made of it in your letter to "Woman's Work," in which you so ably plead the cause of the Zenana Medical College. Few institutions can point to such a band of successful workers as Dr. Griffith can show from his college. Of those who were there with me, three are now working in China, three in India, two in Africa, and two in Syria, all successfully using the knowledge gained there, and seeking to pass it on to others. I have written twice to Dr. Griffith about two of my girls, that I long to get as free pupils into his college. Do you think I have any chance of getting them admitted? I am doing what I can to fit them for it. This year's examination they had eighteen different anatomical questions to write on, and the professor who set them expressed himself much pleased with their answers. This year I will send them down to my Dispensary, which is open twice a week, and let them have some practice in dispensing and seeing patients — LOUISA PROCTOR."

MASSAGE.—In the "Nursing Mirror" for October 6th, a short notice of Massage appears; but the writer wisely says, "It is an operation to be seen, not read about." I would add, "to be most thoroughly studied and cautiously practised." The reason why such prejudice exists against most forms of manipulated treatment seems to me due to the fact that they have been allowed to drift into the hands of relatively ignorant operators. On the Continent and in America physicians do not consider it *infra dig.* to themselves apply massage and electricity, and the trained touch and eye regulate to a nicety the special movements applicable to individual cases, and the actual force necessary; but in England, with few exceptions, massage is left almost entirely to the discretion of a nurse or attendant who thinks a dozen, or often only six, lessons in massage movements (effleurage, pétrinage, massage à friction, and taponement) qualify her to boldly attack almost any form of disease with a conscientious persuasion that the harder she rubs the more good she will do. Very serious results often follow, and I consider the mere movements bear about the same proportion to the art and science of massage as the alphabet does to our language and literature. At least two years' study of the physiology and pathology of the human frame ought to be insisted on as a preliminary qualification, while also much attention to actual physique is necessary for those wishing to adopt the profession. Increased expense would ensure wider success, and there is always room in the field for the ordinary medical rubber.—MASSEUSE.

ARMY NURSING.—I have just seen in this week's HOSPITAL some information about the Netley sisters, and I should like, with your kind permission, to make a few remarks in addition. I have often seen the inquiries of those anxious to find out how to join that "noble band of workers" answered in your pages by referring the inquirers to the Lady Superintendent at Netley. It may be useful to make known that the only way to procure information is to write to the Under Secretary of State for the War Office, Pall Mall. Inquiries of the Lady Superintendent are very rarely answered. The Director-General, A.M.D., selects the candidate (not necessarily after a personal interview), and after acceptance she is sent to Netley, where the Lady Superintendent reports on her fitness for military nursing during her six months' probation. It is very rarely intimated to a sister on probation that she is unsuitable for the work. I do not know how "familiarity with the routine of army life" can qualify a woman for being a nurse. Of course it may depend on what is meant by the "routine of army life." The daughters of officers have not necessarily got the Queen's Regulations at their fingers' ends. If they had, it would not fit them for making better sick-nurses, though it might prepare them somewhat for grappling with the red-tape which hampers every detail of army nursing. even down to making a poultice. The salary is good: though not so ample as at first sight may appear, when unavoidable expenses connected with this branch of nursing are deducted from it. As to the "actual nursing" being done by the orderlies, this is a delusion. The orderlies do all the rough work, and are never to be found when there is anything to teach them, being either engaged in scrubbing corridors or getting ready for parade. It is true that few of them *care* to learn nursing when the opportunity offers, and some excuse may be found in their energies being taxed in the ways mentioned. Some pleasing exceptions may be met now and then; but it can be laid down as a rule that in the Sister's absence there is no-one to take the place of a reliable-trained staff nurse. A good plan is to train a convalescent patient.—K.

### Vacancies.

**The following vacancies are announced:—**

### Matron.

Colchester Infectious Hospital; £52. Sherburn Hospital Durham; £40.

### Nurses.

| | |
|---|---|
| Bradford Infirmary; £25. | Poplar Hospital for Accidents. |
| County of Cornwall Nurses' Home. | District Hospital, Yeovil; £14. |
| Firs Home, Bournemouth; £20. | Blackheath Institute; £20. |
| Cheltenham Institution. | Arbroath Infirmary; £20. |
| Swansea Nursing Institute; several. | Epsom Cottage Hospital. |
| Nottingham Nursing Institute; £25. | Nursing Institution, 96, 97, 98a, Wimpole Street, London, W., Mr. Wilson has several vacancies for thoroughly-trained Nurses. |
| St. Helie's Institute; several; £25. | |
| Ryde Institute. | |
| Canterbury Institute; two. | |
| Hampshire Nurses' Institute. | Hollond Nursing Institution, Nice. |
| West Bromwich District Hospital; £22. | Chislehurst, Sidcup, and Cray Valley Cottage Hospital. |
| Bolton Infirmary and Dispensary; £30 (head). | Birkdale Trained Nurses' Home; two. |
| Cardiff Infirmary; £24. | |

### District Nurses.

| | |
|---|---|
| Newcastle, Staffordshire. | Ryde Institute. |

### Probationers.

| | |
|---|---|
| Peterborough Infirmary. | Bristol Royal Infirmary. |
| Arbroath Infirmary. | Children's Hospital, Birmingham. |
| Stamford, Rutland, and General Infirmary; £16. | Boston Hospital, Lincolnshire. |
| | Macclesfield Infirmary. |

### Notes and Queries.

TO CORRESPONDENTS.—1. Questions may be written on post-cards. 2. Advertisements in disguise are inadmissible. 3. In answering a query please quote the number. 4. If a private answer is desired, a stamped addressed envelope must be forwarded.

Will "Medicus" kindly send name and address, not necessarily for publication?

### Queries.

(76) *The Gate Porter and Female Visitors.*—Reader writes: "A gate porter very roughly put a female visitor out of the gate for giving a slight offence. Is such treatment justifiable, as the Committee do not think he exceeded his duty?"—Without all the facts, it is of course impossible to give an opinion.

### Answers.

*Medical Dictionary.*—We should advise K. G. A. to get "Quain's Dictionary," Churchill; 37*s.* "Hoblyn's Dictionary" is cheaper and smaller, but not so full.

(72) *Blind Asylum.*—Douglas should apply to the Secretary, Association for the Welfare of the Blind, 28, Berners Street, London.

(73) *The Queen's Jubilee Nurses.*—Miss Jackson should write to Sir Rutherford Alcock, K.C.B., Great Queen Street, Westminster, S.W.

### Notice.

THE Doll Show at the Hospital for Sick Children, Great Ormond Street, has been put off till November 9th and 10th.

